# First-Time Parents’ Bonding with Their Baby: A Longitudinal Study on Finnish Parents during the First Eight Months of Parenthood

**DOI:** 10.3390/children10111806

**Published:** 2023-11-14

**Authors:** Jessica Toivo, Noora Tulivuo, Mitsuko Kanzaki, Anna-Maija Koivisto, Jari Kylmä, Eija Paavilainen

**Affiliations:** 1 Unit of Health Sciences, Nursing Science, Faculty of Social Sciences, Tampere University, 33014 Tampere, Finland; jessica.toivo@tuni.fi (J.T.); noora.tulivuo@pirha.fi (N.T.);; 2 Faculty of Nursing, Kyoto Tachibana University, Kyoto 607-8175, Japan; kanzaki@tachibana-u.ac.jp; 3 Unit of Health Sciences, Faculty of Social Sciences, Tampere University, Kalevantie 4, 33014 Tampere, Finland; anna-maija.koivisto@tuni.fi; 4 Etelä-Pohjanmaa Welfare County, 60220 Seinäjoki, Finland

**Keywords:** first-time parent, infant, parental bonding, postpartum depression, Mother-to-Infant Bonding Scale, EPDS, longitudinal study

## Abstract

Early positive bonding between parents and babies promotes the development of parenting skills and parents’ sensitivity to their infant’s needs. Positive bonding has been suggested to decrease the risk of maltreatment. There is less research into the differences between primiparae’s and their spouses’ bonding with their baby and changes in the parent-to-infant bonding during the first year of the baby’s life. The aim of this study was to describe bonding with one’s baby and related differences and changes within first-time parents. The data were collected from nine maternal health clinics in 2019–2021 in one city in Finland. The Mother-to-Infant Bonding Scale (MIBS) and the Edinburgh Postnatal Depression Scale (EPDS) were used. The data were collected during pregnancy (T1) and when the baby was aged 1–2 months (T2) and 6–8 months (T3). The questionnaire was completed separately by the primiparae (*n* = 81 at T1) and their spouses (*n* = 79 at T1). The findings demonstrated that both parents had positive feelings for their baby. The primiparae’s and their spouses’ MIBS scores were relatively low at T2 and T3. The change between time points or the difference in the parents’ bonding was not statistically significant when examining MIBS total scores. The present study identified a positive weak-to-moderate correlation between the MIBS and EPDS. This association was highlighted in the group of primiparae. The results of this study can be used to develop maternity and child health clinic services, and to promote parents’ equal growth in parenthood.

## 1. Introduction

The relationship between parents and babies begins to form during pregnancy as parents develop thoughts, expectations, and feelings toward their unborn child, which initiates bonding between parents and babies. Even though the relationship already begins to form during pregnancy, the bond between parents and babies becomes more tangible after birth [1]. Parental emotional involvement with their baby has been recognized and named as “bonding” [2]. According to Kinsey and Hupcey (2013), bonding illustrates maternal feelings and emotions toward one’s infant [3]. The first months after birth are a highly sensitive period for the development of the bonding between parents and their infant [4], and it has been found to predict the later relationship between babies and parents [5]. However, bonding improves during the first months of a child’s life [2,6].

Parenthood brings joy and happiness but also responsibility and a new role to adapt to. Growth in parenthood is unique and can be challenging [7,8,9]. Although most parents develop a positive bond with their baby, it is common for parents to simultaneously experience opposite emotions [10,11,12]. According to Alves et al. (2021), mothers experience positive and negative feelings toward their babies, such as fear of the unknown, anxiety, joy, and love, among other emotions [10]. Pregnancy and childbirth can be considered empowering [13], and maternity brings a completely new experience of unconditional love and a deep emotional bond that can be experienced only with one’s child [7]. In addition to mothers, fathers have also been found to experience negative emotions during the postpartum period, including anger, touchiness, and resentment of the child [14]. One-third of fathers are overburdened with worries about their parenthood and concern about managing the demands of fatherhood [15]. Bonding between fathers and their children has been studied mainly in Asia. The bonding between fathers and their baby has a positive correlation with the role of the fathers [16]. Fathers’ feelings for their baby are mostly positive [17].

Early positive bonding between parent and their baby promotes the development of parenting skills and parents’ sensitivity to their infant’s needs [18] and promotes the child’s socio-emotional development [19,20,21,22,23,24]. It is suggested that negative bonding such as dislike, distress, or hatred of one’s child might be linked to a risk of maltreatment or child abuse [6,25]. 

Transitioning to parenthood increases the susceptibility of both parents to psychological disorders such as depression [26]. Mothers, compared to fathers, reported more postpartum anxiety, depression, and parenting stress [27,28]. The Edinburgh Postnatal Depression Scale (EPDS) is commonly used in the screening of depressive symptoms [29], and a positive correlation has been found between the EPDS and parental bonding [2]. According to several studies, maternal postpartum depression decreases bonding with one’s baby [30,31,32,33,34,35,36]. Mothers with high scores on the EPDS ten days after delivery showed less intimacy, warmth, and confidence toward their baby during the first year of life [37]. The association between postpartum depression and bonding has been found to occur in both parents [38]. Up to 10% of first-time fathers experience depressive symptoms during their first year of parenthood [39]. It has been found that fathers’ symptoms of depression are related to more negative parenthood and lower affection for one’s baby [40,41,42]. However, according to the literature, average parents have a high level of affection for their babies [2,6,17,34,35,36,43,44].

Primiparae and multiparae have different experiences of parenthood. First-time mothers feel insecure when caring for their babies [10,11,45], and have more negative emotions toward their baby than those with previous deliveries [46]. On the other hand, it has been also found that babies play a more central role in primiparae’s than multiparae’s thoughts [47]. Tsuchida et al. (2019) found that bonding with one’s baby is better at the birth of the second child compared to the first child [35]. However, Cataudella et al. (2022) and Liu et al. (2022) revealed that primiparae have better bonding compared to multiparae [48,49]. Also, it has been found that first-time mothers have more depressive symptoms than mothers with previous children [35,50]. However, according to Tyni et al. (2013), the lives of first-time mothers with depressive symptoms revolve more around their baby compared to those of mothers without symptoms of depression [47].

As the birth of a baby transforms the lives of both parents, it is important to examine the experiences of both parents. Existing research has focused mainly on parturients, while the experiences of spouses regarding parenting and affection for their baby have been less studied. In recent years, parent-to-infant bonding has been studied in Asia, but due to cultural differences, the findings cannot be easily transferred to the Nordic context. There is less research into the differences in mothers and their spouse’s feelings and emotions for their baby, and changes in parent-to-infant bonding during the first year of the baby’s life. Therefore, the present study aimed to describe bonding and related changes between first-time parents with singleton pregnancies and their babies. The research questions were as follows: (1) how do parents rate their bonding with their 1–2-month-old baby? (2) how do parents rate their bonding with their 6–8-month-old baby? (3) what changes occur in the parent–baby bonding between these time points? (4) what are the differences in parents’ bonding with their baby? and (5) how are depressive symptoms associated with bonding with one’s baby? The results of this study can be used to develop child health clinic services, to build parents’ skills for providing sensitive and affirming care for their baby, and to promote equal growth in parenthood. This study was carried out partly during COVID-19 pandemic, which brings forward important aspects concerning parental bonding during isolation measures and cuts of services as well. 

## 2. Materials and Methods

### 2.1. Participants

The data were collected from a convenience sample of primiparae and their spouses, regardless of their biological sex, in nine maternal health clinics in the period 2019–2021 in one relatively large city in Finland. This study was based on questionnaires completed separately by primiparae and their spouses during pregnancy (T1) and when their baby was aged 1–2 (T2) and 6–8 months (T3). Couples with multiple pregnancies were excluded from the study sample. 

The participants provided informed consent at the beginning of the study. The ethics committees of the responsible clinics approved the study before data collection. The term ‘first-time parents’ will be hereinafter used to refer to both parents together; the terms ‘primipara’ and ‘spouse’ will be used to refer to them separately.

### 2.2. Procedures

This study is part of an international longitudinal study carried out in Finland and Japan. In this study, the data collection during pregnancy (T1) included sociodemographic questions (age, education level, family style, employment status, gestational length, and income) and the EPDS. The second (T2) and the third (T3) data collection periods included the EPDS and the Mother-to-Infant Bonding Scale (MIBS). 

This study was introduced to first-time parents by a public health nurse at maternal health clinics. A paper questionnaire was distributed to interested participants. Participants at T1 were given access to an online survey (hosted by REDCap^®^) at T2 and T3. The questionnaires were coded so that the researchers could combine the responses of each family at different time points. 

### 2.3. MIBS

The MIBS is a self-report instrument originally developed for mothers, and it has also been previously used for assessing fathers [17]. Several versions of the MIBS have been developed, and the 10-item version of the MIBS was used in this study. The instrument items are rated on a four-point response scale ranging from 0 to 3, with a total score of 0–30. Some MIBS items are reversed scored. To the best of our knowledge, while no exact cut-off point has yet been determined [6], a high score, nonetheless, indicates worse parent-to-infant bonding. This scale has been found to be easy to respond to [51]. The items of this instrument are presented in Section 3.

Yoshida et al. (2012) reported a two-factor structure for the Japanese version of the MIBS (MIBS-J), which has been used in subsequent studies [6]. The MIBS-J has two subscales: Lack of Affection (LA) (items 1, 6, 8, and 10, Cronbach’s alpha = 0.71) and Anger and Rejection (AR) (items 2, 3, 5, and 7, Cronbach’s alpha = 0.51), both of which range from 0 to 12 [6]. It is crucial in clinical practice to understand both subscale scores and the total score to ensure that no parent in need of support is overlooked [52]. Therefore, both total scores and subscale scores were analyzed and reported here. The reliability and validity of the MIBS-J has already been confirmed [6,52]. 

The 10-item version of the MIBS has not been used in Nordic countries before. The MIBS was translated by a native Finnish translator, and a back translation was completed by a native Japanese translation specialist. Subsequently, the instrument was confirmed and modified by two nursing researchers, resulting in the final version. 

### 2.4. EPDS

The EPDS was used to evaluate the presence of depressive symptoms, reflecting parents’ experiences in the previous seven days. Although the EPDS was originally designed to screen for postnatal depression, it is also a feasible questionnaire for use beyond the postpartum period. The EPDS is a 10-item self-report instrument, where each item is rated on a four-point Likert scale ranging from 0 to 3, with a total score of 0–30 (Cronbach’s alpha = 0.78). A higher total score indicates more severe depression [53,54]. In Finland, the recommended cut-off point to screen for major depressive disorder (10 or more) is used to differentiate the group of mothers at risk of significant depressive symptoms [55,56]. The EPDS has also been found to be a reliable and valid tool for measuring fathers’ mood. However, screening for depression or anxiety disorders in fathers has a lower cut-off of two points than screening in mothers [57].

### 2.5. Statistical Analysis

The data were analyzed using SPSS Statistics 28.0. The sum variables were formed in line with the instructions of the instruments. The total score of the MIBS was formed by adding the values obtained on all items, and scores for the LA and AR subscales were formed separately. Also, the total score of the EPDS was calculated. The internal consistency of each scale was verified using Cronbach’s alpha coefficients (Table 1), although the values remained at a rather low level.

With the exception of age, the data for the other variables were found to deviate from a normal distribution. As a result, the median values (Md) and the lower (Q1) and upper quartiles (Q3) were reported. Statistical testing was undertaken using non-parametric Wilcoxon tests to examine the differences between primiparae’s and spouses’ parental bonding, and the changes in parental bonding between time points, separately for each group. The association between the MIBS and EPDS was examined via Spearman’s rank correlation. Correlation coefficients between 0.1 and 0.3 were considered as representing a weak relationship between the variables, from 0.3 to 0.5 as moderate, and >0.5 as strong. *p*-values < 0.05 were considered statistically significant [58].

## 3. Results

### 3.1. Participants

The response rate at T1 was 35.6%. The respondents were primiparae and their spouses, and all represented the family type of a nuclear family (100%). The primiparae (*n* = 81) were 29.8 years old on average (SD = 4.0), with the youngest being 21 years old and the oldest being 39 years old. The spouses (*n* = 78) were 32.1 years old on average (SD = 5.9), with the youngest being 21 years old and the oldest being 54 years old. More than half of the primiparae (50.6%) had a university-level education. Respectively, one third of the spouses (33.0%) had the same level of education. The majority of the primiparae (62.5%) and their spouses (88.5%) were employed. Almost one third of the primiparae (28.8%) reported that they were not currently employed, and almost one tenth (8.8%) reported themselves as being unemployed. The respondents who reported not being currently employed included students or those on sick leave. The background factors of the participants are presented in Appendix A.

### 3.2. Parents’ Bonding with Their Baby

The MIBS total scores and subscale scores of both primiparae and spouses at both time points were relatively low (Table 2). The parents experienced mainly positive bonding with their baby (Table 3).

### 3.3. Differences and Changes in Parental Bonding 

When examining the differences in parental bonding between primiparae and their spouses, no statistically significant difference was found in the parents’ bonding with their baby at either of the studied time points (Table 4).

When examining changes in the parents’ bonding between time points, a statistically significant change was observed in the AR subscale scores for both parents. This indicated that feelings of anger and rejection decreased between the two time points in the group of primiparae (*p* = 0.050) and the group of spouses (*p* = 0.048). Overall, the average scores of parental bonding declined slightly between the time points, although the change was not statistically significant. This indicated that parental bonding with the baby improved as the baby grew (Table 4).

### 3.4. The Association between MIBS and EPDS

As shown in Table 5, nearly all correlations between the MIBS and EPDS were positive. In the group of primiparae, the correlation between MIBS total scores at T2 and EPDS scores at T1 (r = 0.427) and T2 (r = 0.395) was positive and moderate. When examining the association between the LA subscale and EPDS in the group of primiparae, there was a moderate correlation between LA scores at T2 and EPDS scores at T1 (r = 0.400) and T2 (r = 0.360). In the group of primiparae, there was a moderate correlation between the AR subscale at T2 and the EPDS at T1 (r = 0.314) and at T2 (r = 0.327), respectively. In the group of spouses, the correlation between AR scores at T2 and EPDS scores at T2 was positive and moderate (r = 0.318).

## 4. Discussion

In this study, the average age of the primiparae was 30 years and the average age of their spouses was 32 years, which is similar to national data. The analytical sample was limited to two-parent households, which should be considered when assessing the transferability of the results. A study of families with a baby conducted in Finland found that up to one third of parturients and 13% of spouses had depressive symptoms during pregnancy. Among parturients, 15% suffered from postpartum depressive symptoms [59]. In this study, the average EPDS score was relatively low. In the group of primiparae, only 8.6% scored above the EPDS cut-off point (ten or more) during their pregnancy. In the group of spouses, the cut-off point was eight or more, and nearly 9% reached it during pregnancy. When measured at T2, about 15% of primiparae and spouses reached the EPDS cut-off point, and at the final time point, the rate was nearly 7% in the group of primiparae and about one in ten in the group of spouses. The special features of Finnish maternity care services should be considered when making international comparisons, as these may have contributed to the primiparae’s and their spouses’ growth into parenthood. In Finland, primiparae are offered at least nine health examinations at a maternal health clinic, and a public health nurse’s home visit during pregnancy and another after childbirth. Both parents are invited to these health appointments. The family’s well-being continues to be monitored at a child health clinic until the child reaches school age [60]. 

To the best of our knowledge, this study is the first to examine both parents’ bonding with their infant and the change therein during the first eight months of the child’s life using the Mother-to-Infant Bonding Scale in Finland. As shown in previous studies [2,6,17,35,36,43,44], the results showed that the parents had a strong affection for their baby. A majority of the parents felt love toward their baby and felt close to their baby. The parents wanted to protect and care for their baby. The two-factor structure derived by Yoshida (2012) was used in this study [6]. Taking into consideration the two-factor structure, the bond between the parents and their baby was strong. The LA factor describes parents’ lack of affectionate and caring attitudes toward the baby, i.e., a high LA score suggests indifference and loss of enthusiasm in caring for the baby. Meanwhile, the AR factor describes parental anger and negative feelings toward the baby. These bonding subscales can give more evidence of parents’ feelings toward their baby and indicate possibilities to offer parents more necessary support to prevent child maltreatment [6]. This issue should also be studied using larger data set and in more detail in Finland to obtain more information on the internal consistency of the MIBS and its usefulness in measuring parental bonding.

Transition to parenthood begins to form during pregnancy and continues after birth [1,48]. Growth in parenthood is unique [6,8,9], and several studies have shown that many factors are associated with parent–infant bonding. Parents’ positive attitudes and realistic expectations about parenthood during pregnancy promote bonding [18,61], and parents’ personality traits and previous experiences have an association with bonding [16,42,62,63,64]. It has been found that mental health symptoms [17,27,34,38,42,44,51,63,65,66,67,68,69,70] and the quality of parents’ intimate relationship [27,34,40,42,48,63,66,70] are associated with bonding. In addition, parents’ socio-economic status has been found to be associated with bonding [17,40,67,71]. Also, a baby’s characteristics, such as illness, a difficult temperament, or sleep disorders, may challenge the bonding [20,27,34,51]. Physical and emotional proximity between parents and babies promotes bonding [40,67,72,73,74]. Fijałkowska and Bielawska-Batorowicz (2020) identified that the determinants of parental attachment differ between genders [75]. 

The results of our study add to the current body of literature and confirm the findings reported by Partfitt et al. (2014), Taylor et al. (2005), and Yoshida et al. (2012), indicating that parental bonding develops progressively, which is comparable to our results [2,6,27]. Parents’ bonding with their baby improves during the first eight months of the child’s life. Longitudinal research on the development of both parents’ bonding with their baby remains scarce, and therefore, more research evidence is needed. 

Parents’ bonding with their baby tends to be similar [73], indicating no differences between the sexes. However, some studies [76,77] have found that mothers have better bonding scores, indicating less concern for a lack of postpartum bonding among mothers compared to fathers, whereas Ngai and Lam (2023) found that mothers have more difficulties in bonding with their baby compared to fathers [38]. Based on the data of this study, it is not possible to confirm or deny the difference between the primiparae’s and spouses’ bonding with their baby. 

Vismara et al. (2016) reported that mothers feel more anxiety, depression, and parenting stress than their spouses [28]. In a study conducted in Japan, Kasamatsu et al. (2019) found that postpartum depression may be a strong predictor of disorders in mother–to–infant bonding during the baby’s first year [44]. In the present study, there was a moderate association between bonding when the baby was 1–2 months old and the primipara’s depressive symptoms during pregnancy and during the first months of the baby’s life. This association between symptoms of depression and bonding in the group of spouses was weak but positive, except for the association between the anger and rejection factor and the EPDS when the baby was 1–2 months old, which was positive and moderate. This supports the assumption that, as the depression risks of parents increase, their attachment levels decrease. This finding is consistent with previous studies that have found an association between mother–infant bonding and depression [5,19,30,35,36,38,44,78]. According to Badr et al. (2018), depressed mothers have trouble adapting to parenthood, and they are less sensitive to the needs of their baby, leading to weaker bonding with the baby [78]. Therefore, our findings suggest that primiparae who tend to be depressed during pregnancy may need support to reduce depression or strengthen their bonding with their baby within the early postpartum period.

### Limitations

There are some limitations in this study. This study was conducted in a relatively large city in southern Finland. The results could be different if the study had been conducted in a rural municipality. The data for this study were collected in 2019–2021 during the global COVID-19 pandemic. According to previous studies, the pandemic has reduced parental preparedness for those expecting their firstborn [79] and affected the well-being of families with children [80,81], and COVID-19-related stress, grief, and health worries have also been found to be associated with mother–infant bonding [49,64]. This poses a limitation to the transferability of the results of this study, as every set of data is a reflection of its own time. 

Moreover, it is essential to consider the group of non-participants. The survey was part of a larger research project and the extensive length of the questionnaire might have reduced some potential participants’ readiness to participate in the study. It is very likely that those with more eager attitudes toward parenting or a better parental mental status responded. The survey was carried out at a time when first-time parents were faced with a new phase of their lives and might have felt burdened with their everyday lives, and for this reason, they might have dropped out of the follow-up data collection. The small sample size also limits the ability to make broader generalizations of the findings. 

Caution should also be exercised because the parents self-assessed their emotions. It has been suggested that self-assessment questionnaires are more susceptible to social desirability bias [82]. However, online surveys may be an appropriate alternative for detecting possible variation in responses to the MIBS due to a decrease in desirability bias and an increased perception of anonymity [83]. The self-report MIBS has been found to work well at detecting parents’ bonding with their baby, which cannot be externally assessed through observations [51]. 

Lastly, the Mother-to-Infant Bonding Scale and the EPDS are used internationally and have been found to be valid. Nevertheless, the validity of the MIBS has not been verified in the Finnish socio-cultural environment. Due to the small sample size, it is not possible to confirm the validity of the MIBS in the present study. When inspecting the Cronbach’s alpha, the internal consistency of the MIBS remained low, but this could be explained by the homogeneity of the respondents in their responses [84]. Moreover, due to the small sample size, it was not possible to perform a factor analysis, which could have led to finding more detailed research evidence on the LA and AR subscales.

Notwithstanding the above limitations, the present study has several strengths. In particular, it is one of the first to focus on investigating Finnish primiparae’s and their spouses’ bonding with their baby, and the differences in bonding between parents. Secondly, this is a longitudinal study, which investigated both continuity and change in variables across two time points.

## 5. Conclusions

The analysis demonstrated that both parents had positive feelings toward their baby. Furthermore, this study found that parental bonding improved as the baby grew. The present study identified a positive correlation between the MIBS and EPDS. This association was highlighted in the group of primiparae. If there are challenges in bonding or parents have mental health issues, it is crucial that they get the help and support they need to improve the situation within their family. Health and social care professionals need to have the knowledge and skills to identify bonding difficulties and support parents. If needed, multi-professional expertise should also be provided.

### Future Directions 

The validation of the MIBS was not performed in this study due to the small sample size, so validating the MIBS in the Finnish context in the future is recommended.

The participants in our sample had no significant psychological challenges and demonstrated appropriate parental affection and positive bonding with their babies. Future studies including also parents experiencing mental stress could lead to a wider distribution of scores.

Parents living in one-parent families have been found to face more challenges in coping than parents living in nuclear families [85,86,87]. In the future, it will be necessary to examine whether bonding is different in parents living in one-parent families than in those living in nuclear families.

## Figures and Tables

**Table 1:** Cronbach’s alphas (α) for MIBS, MIBS subscales, and EPDS.

Scale	Primiparae (α)			Spouses (α)		
	T1	T2	T3	T1	T2	T3
MIBS		0.679	0.547		0.492	0.485
LA		0.702	0.691		0.460	0.304
AR		0.438	0.427		0.152	0.196
EPDS	0.832	0.886	0.876	0.787	0.893	0.822

MIBS = Mother-to-Infant Bonding Scale total score; LA = Lack of Affection subscale score; AR = Anger and Rejection subscale score; EPDS = Edinburgh Postnatal Depression Scale total score. T1 = during pregnancy; T2 = 1–2 months postpartum; T3 = 6–8 months postpartum.

**Table 2:** Parental bonding at T2 and T3.

		*n*	Md	Q_1_–Q_3_
Primiparae’s MIBS at T2		62	2	1.00–2.25
	LA at T2	62	0	0.00–1.00
	AR at T2	62	1	1.00–2.00
Spouses’ MIBS at T2		53	2	1.00–3.00
	LA at T2	53	1	0.00–1.00
	AR at T2	53	1	0.50–2.00
Primiparae’s MIBS at T3		58	1	0.00–3.00
	LA at T3	58	0	0.00–1.00
	AR at T3	58	1	0.00–2.00
Spouses’ MIBS at T3		42	1	0.75–3.00
	LA at T3	42	1	0.00–1.00
	AR at T3	43	1	0.00–1.00

*n* = The data for all respondents at T2 or T3 were analyzed. Md = median; Q_1_ = lower quartile; Q_3_ = upper quartile. MIBS = Mother-to-Infant Bonding Scale total score, range 0–30; LA = Lack of Affection subscale score, range 0–12; AR = Anger and Rejection subscale score, range 0–12.

**Table 3:** Parents’ feelings and emotions toward their baby.

		Primiparae (T2 *n* = 62, T3 *n* = 58)				Spouses (T2 *n* = 53, T3 ^1^ *n* = 43)			
		Very Much So, Most of the Time	Very Much So, Some of the Time	Slightly, Some of the Time	Not at All	Very Much So, Most of the Time	Very Much So, Some of the Time	Slightly, Some of the Time	Not at All
Items Describing Bonding with the Baby (MIBS)	Time Point	%	%	%	%	%	%	%	%
1. I feel loving towards my baby	T2	93.5	6.5			94.3	3.8		1.9
	T3	94.8	3.4	1.7		90.7	7.0		2.3
2. I feel scared or panicky when I have to do something for my baby	T2		3.2	66.1	30.6		7.5	54.7	37.7
	T3	3.4		46.6	50.0		4.7	39.5	55.8
3. I feel resentful towards my baby	T2			43.5	56.5			30.2	69.8
	T3			41.4	58.6			25.6	74.4
4. I feel nothing for my baby	T2			6.5	93.5			9.4	90.6
	T3	1.7		5.2	93.1		2.3	7.0	90.7
5. I feel angry with my baby	T2				100			5.7	94.3
	T3		1.7		98.3				100
6. I enjoy doing things for my baby	T2	69.4	27.4	3.2		49.1	45.3	5.7	
	T3	65.5	31.0	3.4		50.0	38.1	11.9	
7. I wish my baby was different	T2			16.1	83.9		1.9	5.7	92.5
	T3			6.9	93.1		2.3	2.3	95.3
8. I feel protective towards my baby	T2	95.2	4.8			98.1	1.9		
	T3	96.6	1.7	1.7		100			
9. I wish I did not have my baby	T2			1.6	98.4			1.9	98.1
	T3			1.7	98.3			4.7	95.3
10. I feel close to my baby	T2	91.9	6.5	1.6		83.0	15.1	1.9	
	T3	91.4	8.6			93.0	7.0		

T2 = 1–2 months postpartum; T3 = 6–8 months postpartum; ^1^ = item 6, *n* = 42.

**Table 4:** Differences and changes in parental bonding.

Difference in Scores between Primiparae and Spouses		*n*	Primiparae		Spouses		*p*
			Md	Q_1_–Q_3_	Md	Q_1_–Q_3_	
	MIBS at T2	51	2	1.00–3.00	2	1.00–3.00	0.528
	LA at T2	51	0	0.00–1.00	1	0.00–1.00	0.069
	AR at T2	51	1	0.00–2.00	1	0.00–2.00	0.299
	MIBS at T3	40	1	0.00–3.00	1	0.25–3.00	0.670
	LA at T3	40	0	0.00–1.00	0,5	0.00–1.00	0.061
	AR at T3	41	1	0.00–2.00	1	0.00–1.00	0.266
**Change in scores between T2 and T3**		***n***	**T2**		**T3**		***p***
			**Md**	**Q_1_–Q_3_**	**Md**	**Q_1_–Q_3_**	
	Primiparae’s MIBS	58	2	1.00–2.25	1	0.00–3.00	0.319
	Primiparae’s LA	58	0	0.00–1.00	0	0.00–1.00	0.415
	Primiparae’s AR	58	1	1.00–2.00	1	0.00–2.00	0.050
	Spouses’ MIBS	42	2	1.00–3.00	1	0.75–3.00	0.198
	Spouses’ LA	42	1	0.00–1.00	1	0.00–1.00	0.843
	Spouses’ AR	43	1	1.00–2.00	1	0.00–1.00	0.048

Md = median; Q_1_ = lower quartile; Q_3_ = upper quartile. MIBS = Mother-to-Infant Bonding Scale total score; LA = Lack of Affection subscale score; AR = Anger and Rejection subscale score.

**Table 5:** Associations between parents’ MIBS and EPDS scores.

Primiparae	MIBS					
	T2 (*n* = 62)			T3 (*n* = 58)		
	Total	LA	AR	Total	LA	AR
EPDS during pregnancy (T1)	r = 0.427 *p* < 0.001	r = 0.400 *p* = 0.001	r = 0.314 *p* = 0.013	r = 0.249 *p* = 0.059	r = 0.271 *p* = 0.040	r = 0.168 *p* = 0.207
EPDS at T2	r = 0.395 *p* < 0.001	r = 0.360 *p* = 0.004	r = 0.327 *p* = 0.009	r = 0.240 *p* = 0.069	r = 0.257 *p* = 0.051	r = 0.237 *p* = 0.073
EPDS at T3				r = 0.273 *p* = 0.038	r = 0.144 *p* = 0.279	r = 0.279 *p* = 0.034
**Spouses**	**MIBS**					
	**T2 (*n* = 53)**			**T3 (*n* = 42)**		
	**Total**	**LA**	**AR**	**Total**	**LA**	**AR**
EPDS during pregnancy (T1)	r = 0.126 *p* = 0.369	r = 0.082 *p* = 0.559	r = 0.168 *p* = 0.228	r = 0.047 *p* = 0.767	r = 0.013 *p* = 0.936	r = −0.058 ^1^ *p* = 0.710
EPDS at T2	r = 0.298 *p* = 0.030	r = 0.231 *p* = 0.096	r = 0.318 *p* = 0.020	r = 0.224 *p* = 0.153	r = 0.145 *p* = 0.360	r = 0.231 ^1^ *p* = 0.136
EPDS at T3				r = 0.287 *p* = 0.066	r = 0.201 *p* = 0.202	r = 0.213 ^1^ *p* = 0.170

r = Spearman’s rank correlation coefficient; ^1^ *n* = 43.

## Data Availability

Data are contained within the article.

## Appendix A

**Table A1:** The background factors of participants.

	*n*	%	Mean (SD) ^1^ or Md (Q1–Q3) ^2^
**Age of the primiparae (years)**	81		29.8 (4.0) ^1^
**Age of the spouses (years) ^3^**	78		32.1 (5.9) ^1^
**Family style reported by the primiparae: nuclear family**	81	100.0%	
**Educational level of the primiparae**			
Basic education or vocational training	19	23.5%	
College-level education	21	25.9%	
University-level education	41	50.6%	
**Educational level of the spouses**			
Basic education or vocational training	25	32.1%	
College-level education	27	34.6%	
University-level education	26	33.3%	
**Employment status of the primiparae**			
Unemployed	7	8.8%	
Employed	50	62.5%	
Not currently working	23	28.8%	
**Employment status of the spouses**			
Unemployed	3	3.8%	
Employed	69	88.5%	
Not currently working	6	7.7%	
**Annual income of the primiparae (EUR)**			
≤20,000	18	22.5%	
20,000–40,000	41	51.2%	
40,000–80,000	21	26.3%	
**Annual income of the spouses (EUR)**			
≤20,000	11	14.5%	
20,000–40,000	29	38.2%	
40,000–80,000	32	42.1%	
≥80,000	4	5.3%	
**Gestational length**			
2nd trimester	13	16.0%	
3rd trimester	68	84.0%	
**EPDS of the primiparae**			
T1	81		5 (2.5–7) ^2^
T2	62		5 (2–7) ^2^
T3	58		5 (3–7.25) ^2^
**EPDS of the spouses**			
T1	79		3 (2–5) ^2^
T2	53		3 (1–5.5) ^2^
T3	43		2 (1–5) ^2^

^1^ = Mean (SD); ^2^ = Md (Q1–Q3); ^3^ = only ages over 15 years considered.
